# Preliminary Report on the Effect of Savanna Plants *Leucaena leucocephala*, *Parkia platycephala* and *Senna alata* against Eggs and Immature Stages of Trichostrongylid Nematodes In Vitro

**DOI:** 10.3390/pathogens9120986

**Published:** 2020-11-26

**Authors:** Benta Natânia Silva Figueiredo, Marcello Otake Sato, Laiane Teixeira Sousa Moura, Sandra Maria Botelho Mariano, Tarso da Costa Alvim, Ilsamar Mendes Soares, Satoru Kawai, Sergio Donizeti Ascêncio, Helcileia Dias Santos, Joseilson Alves Paiva, Megumi Sato, Viviane Mayumi Maruo

**Affiliations:** 1 Escola de Medicina Veterinária e Zootecnia, Universidade Federal do Tocantins, Araguaína 77804-970, Tocantins, Brazil; benta.figueiredo@catolica-to.edu.br (B.N.S.F.); laiane.moura@uft.edu.br (L.T.S.M.); hdsantos@mail.uft.edu.br (H.D.S.); paiva@mail.uft.edu.br (J.A.P.); vivimaruo@mail.uft.edu.br (V.M.M.); 2 Escola de Ciências Agrárias e Ambientais, Centro Universitário Católica do Tocantins, Palmas 77061-002, Tocantins, Brazil; 3 Department of Tropical Medicine and Parasitology, Dokkyo Medical University, Mibu 321-0293, Tochigi, Japan; skawai@dokkyomed.ac.jp; 4 Curso de Medicina, Universidade Federal do Tocantins, Palmas 77001-090, Tocantins, Brazil; sandrabotelho@mail.uft.edu.br; 5 Laboratório de Pesquisa em Produtos Naturais, Universidade Federal do Tocantins, Palmas 77001-090, Tocantins, Brazil; tarso@uft.edu.br (T.d.C.A.); ilsamar.soares@ifto.edu.br (I.M.S.); sergioda@mail.uft.edu.br (S.D.A.); 6 Graduate School of Health Sciences, Niigata University, Niigata 951-8518, Niigata, Japan; satomeg@clg.niigata-u.ac.jp

**Keywords:** nematicide activity, parasite control, phytotherapy, plant secondary metabolites

## Abstract

The current study evaluated the anthelmintic effect of different extracts of *Leucaena leucocephala*, *Parkia platycephala,* and *Senna alata* on trichostrongylid eggs and infective larvae and determined the potential active components of each plant. Dried and macerated plant material was concentrated using rotaevaporation to obtain the crude extract (CE), followed by solvent partitioning to obtain hexanic (HexE), acetatic (AcE), and butanolic (BuE) extracts used for phytochemical analysis and anthelmintic efficacy testing in vitro. All the crude and partitioned extracts tested showed inhibition activity in the hatching of trichostrongylid eggs. Larvicidal efficacy was observed at CE concentrations of 2.5, 5.0, and 7.5 mg/mL for *P. platycephala* and *S. alata*. However, *L. leucocephala* CE did not significantly reduce the number of living larvae in the tested concentrations. Chromatographic analysis revealed several active metabolites; gallic acid, ellagic acid, naringin, morin, and kaempferol on AcE of *P. platycephala*; gallic acid, rutin, and ellagic acid on BuE of *P. platycephala*; and gallic acid and naringin on BuE of *L. leucocephala*. The extracts of *P. platycephala*, *L. leucocephala,* and *S. alata* leaves showed egg hatching inhibition and larvicidal activity, probably produced by tannins and flavonoids, which may act alone or by synergism.

## 1. Introduction

Trichostrongylids are the most important nematode parasites of livestock causing gastroenteritis, especially in young animals [1]. The control is usually satisfactorily done by chemotherapy; however, anthelmintic resistance has been observed to the most variety of active principles [2,3,4]. Thus, alternatives in parasite control are necessary to improve animal health and livestock production [5].

Plant extracts have been used to control parasites since ancient times [6]. In Brazil, a continental country, there are remarkable differences in access to medicine in rural areas, especially in remote areas of the Amazonian region, where there is popular knowledge used for treating several diseases, including helminth parasites [7,8]. However, studies on their efficacy are important in order to support the use and the indications for those local medicines.

Different studies on the active principles of plants for the treatment of diseases have been carried out pointing secondary metabolites of plants as tannins and other polyphenols as the effective molecules against parasitic nematodes [6,9,10,11].

*Leucaena leucocephala, Parkia platycephala,* and *Senna alata* are plants of the Brazilian savanna and Amazon ecosystems commonly used for the treatment of helminth infections in animals by local people adding the plants in natura to the forage, but their direct effects on parasites are unknown. To increase the understanding on the anthelmintic plants and to validate the local knowledge, the current study evaluated the anthelmintic effect of different extracts of *L. leucocephala, P. platycephala,* and *S. alata* on trichostrongylids eggs and infective larvae and determined the potential active components of each plant.

## 2. Results

### 2.1. Phytochemical Analysis

The phytochemical analysis of *L. leucocephala* leaves revealed the presence of phenols in HexE and AcE, condensed tannins and saponins in BuE, and flavonoids in AcE.

The *P. platycephala* leave tests showed the presence of hydrolyzed tannins both in AcE and BuE and saponins in AcE and BuE. The phytochemical tests with *S. alata* leaves presented chalcones and aurones in HexE and flavonoids, chalcones, aurones, and phenols in AcE. In the BuE of *S. alata* leaves, phenols, flavonoids, flavones, flavonols, xanthones, and saponins were detected.

**Figure 1:**
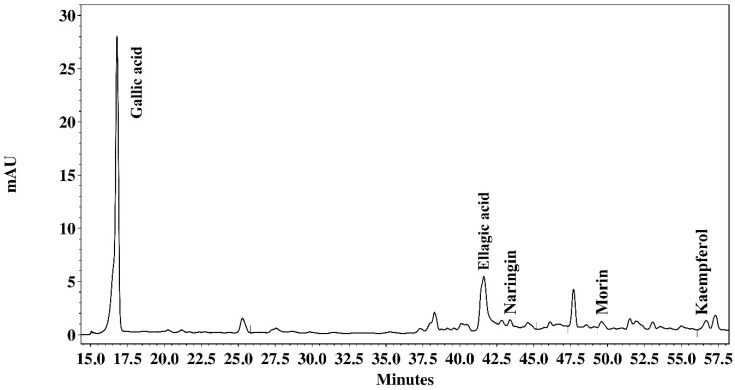
Milli-absorbance (mAU) detection at 254 nm showing the HPLC fingerprinting of acetatic extract of *P. platycephala*.

**Figure 2:**
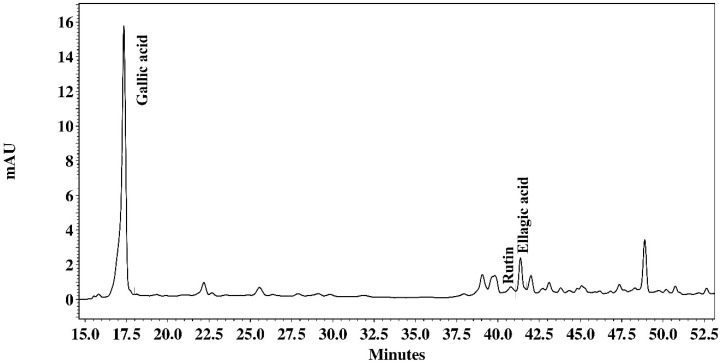
Milli-absorbance (mAU) detection at 254 nm showing the HPLC fingerprinting of butanolic extract of *P. platycephala*.

**Figure 3:**
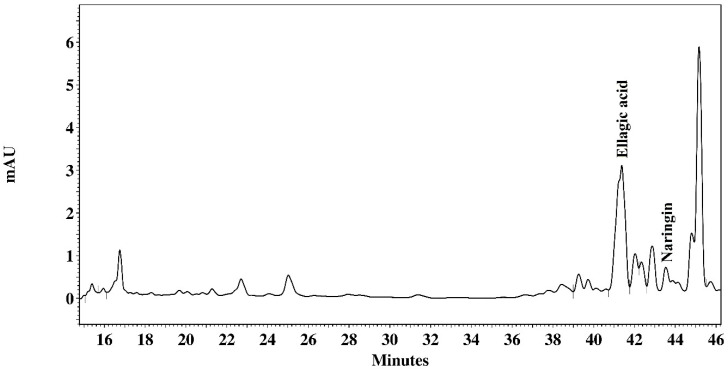
Milli-absorbance (mAU) detection at 254 nm showing the HPLC fingerprinting of butanolic extract of *L. leucocephala*.

The chromatographic analysis of the AcE fraction of *P. platycephala* detected the presence of gallic acid (136.246 µg/mL), ellagic acid (19.368 µg/mg), naringin (7935 µg/mg), morin (1602 µg/mg), and kaempferol (3341 µg/mg) (Figure 1). The BuE fraction of *P. platycephala* detected the presence of gallic acid (51.050 µg/mg), rutin (7.892 µg/mg), and ellagic acid (10.029 µg/mg) (Figure 2). The BuE of *L. leucocephala* detected the presence of gallic acid (23.975 µg/mg) and naringin (2606 µg/mg) (Figure 3).

### 2.2. The Egg Hatching Inhibition Effect of the Savanna Plant Extracts

The results of EHIs using crude and partitioned extracts of *L. leucocephala, P. platycephala,* and *S. alata* showed all the plant extracts tested in this study presented significant inhibition in the hatching of eggs of trichostrongylids. EHIs using CE presented significant inhibition from concentrations of 0.5, 1.0 e 1.5 mg/mL of CE of *L. leucocephala, P. platycephala* and *S. alata,* respectively (*p* < 0.05) (Table 1).

The EHI using the CE of *P. platycephala* was effective on inhibiting eggs’ hatching presenting 84.5%, 98.1%, and 99% of inhibition in CE concentrations of 0.5, 1.0, and 1.5 mg/mL, respectively. This activity in the parasite eggs was also observed in BuE of *P. platycephala* with values of EHI of 60.9%, 99.6%, and 100% of inhibition in concentrations of 0.5, 1.0, 1.5 mg/mL, respectively. The results of AcE of *P. platycephala* revealed EHI values of 72.6%, 71.16%, 59.8%, 96.87% and 100% in AcE concentrations of 0.05, 0.1, 0.5, 1.0 and 1.5 mg/mL, respectively (Table 1).

Egg hatching inhibition activity using *L. leucocephala* CE was also observed with EHI values of 90.5%, 92%, and 93.9% in concentrations of 0.5, 1.0 and 1.5 mg/mL, respectively. Partitioned *L. leucocephala* material showed EHIs of 59.4%, 63.63%, 66.24%, 63.64% and 58.78% in the concentrations 0.05, 0.1, 0.5, 1.0 and 1.5 mg/mL of HexE and 30.94%, 38.73%, 65.42% and 97.92% in the concentrations 0.1, 0.5, 1.0 and 1.5 mg/mL of BuE, respectively. The AcE *L. leucocephala* did not show significant differences of activity in eggs hatching comparing with the non-treated control (Table 1).

The CE of *S. alata* presented eggs hatching inhibition in EHI with 93.4%, 95.3%, and 99.3% of inhibition in the concentrations of 0.5, 1.0, and 1.5 mg/mL, respectively. The HexE of *S. alata* showed EHI of 70%, 68.44%, 62.78% and 64.62% in the concentrations 0.1, 0.5, 1.0 and 1.5 mg/mL, respectively. The BuE of *S. alata* did not present significant differences (Table 1).

### 2.3. Effectiveness of Extracts in the Larval Stage

LMTs were performed using CE to examine the possible synergistic action of the plants’ compounds. The tests showed significant effectiveness by CE of *P. platycephala* at concentrations of 2.5, 5.0, and 7.5 mg/mL presenting 23.7%, 53.19% and, 54.98%, of L3 mortality rates respectively, after 72 h of exposure (Table 2). The treatment with CE of *S. alata* leaves revealed significant differences in the concentrations of 2.5, 5.0, and 7.5 mg/mL with L3 mortality rates of 49.71%, 25.8%, and 29.4%, respectively after 48 h exposure and 49.58%, 47.26% and 47.94% in the concentrations of 2.5, 5.0 and 7.5 mg/mL of *S. alata* CE, respectively, after 72 h (Table 3). The CE of *L. leucocephala* did not cause a significant effect on L3 of trichostrongylids in any tested concentration (Table 4).

## 3. Discussion

The CE of *S. alata* presented eggs hatching inhibition and larvicidal effects against trichostrongylids. The phytochemical analysis of *S. alata* leaves revealed the presence of flavonoids, as also described by [12]. Flavonoids are polymers widely distributed in nature, with effects demonstrated on bacteria, fungi, viruses, and endoparasites [13]. However, the partitioned extracts of *S. alata* showed low efficacy on trichostrongylids eggs, possibly because the molecules in the plant promote their action through synergism [14].

Phenolic compounds, found in all plants, are classified as simple phenols, phenolic acids, flavonoids, tannins, and lignans [15]. These molecules can act as antioxidants, as well as antimicrobials, antifungals, and anthelmintics [15,16,17,18]. In the plants of the present study, ellagic acid, gallic acid, kaempferol, and naringin, polyphenols were identified and were described as anthelmintics [19,20,21,22].

Polyphenols are molecules that can neutralize free radicals, besides quench electrons from various biological systems including that of electron transport chain [23]. The uncoupling oxidative phosphorylation can promote depletion of ATP production that might be the possible mechanism of action of these compounds [21].

The gallic acid and ellagic acid were detected in the BuE and AE of *P. platycephala*. Both compounds are major constituents of the hydrolyzable tannins (HT) group [15] and correlated to the nematicide action of *Adocacia indica* and *Mangifera indica* [24,25]. In this sense, EHI and larvicidal activity observed with the extracts of *P. platycephala* leaves may occur by the action of tannins, despite, in this study, it not being possible to determine exactly the determinant molecules.

The presence of phenolic compounds with the anthelmintic action of extracts of seeds and leaves of *P. platycephala* is variable [26] and the solvents and protocols used for extraction promote variation in concentrations and the classes of secondary metabolites present in extracts, as well as the place and period of collection [27].

Only the BuE extract of *L. leucocephala* presented EHI activity and action can be assigned to the condensed tannins (CT) that were identified by colorimetric tests. CT or proanthocyanidins are complex structures of polymers of flavan-3-ol and/or flavan-3,4-diols, which present pattern replacements between flavanic units, diversity of position between their bonds, and stereochemistry of their compounds [28].

Tannins are secondary metabolites that can produce anthelmintic activity through their ability to bind with metals and proteins [18]. The tannins are reportedly capable of promoting adult parasites death, inhibiting egg hatching in vitro, and reducing worm burden in vivo [18,29,30], their action seems to be related to molecular weight [31], to monomers of the molecule involved and to the parasite species tested [32]. However, the antiparasitic effect of tannins is associated with their structure, ingested amount, availability in the gastrointestinal tract, and its concentration as an active principle in the plant, which can vary between different regions and seasons [33].

Inhibitory action was observed using aqueous extract of the seeds of *L. leucocephala* on strongyles infective larvae (L3) of sheep in Nigeria [34]. However, in Brazilian extracts of *L. leucocephala* against *H. contortus*, despite an effective EHI no larvicidal activity was observed. The EHI was apparently due to the activity of proteases and chitinases [35]. Thus, these studies demonstrate that the plant's geographical origin, their parts, and the form of extraction could interfere in the extract activity against helminths in different life stages.

In vitro tests to evaluate the inhibition of egg hatching, larval motility, and adult worm motility are screening tests that allow for the evaluation of antiparasitic activity of medications and the anthelmintic potential of novel products since the compounds are in direct contact with the different life-cycle stages of the parasite [36,37]. According to the reference guideline [38], effective anthelmintic agents should promote inhibition rates of more than 90%; moderately effective agents should promote inhibition from 80 to 90%, and compounds with less than 80% of inhibition are considered of low efficacy. Thus, the CE and the BuE and AE fractions of P. *platycephala,* the CE and BuE of *L. leucocephala,* and CE of *S. alata* showed to be effective anthelmintic agents promoting inhibition of egg hatching of more than 90%.

## 4. Materials and Methods

### 4.1. Plant Material

The plants were collected in Araguaína city, from September of 2012 to January of 2013, during the rainy season. The collected species were identified, deposited, and registered in the Herbarium of Federal University of Tocantins as *L. leucocephala* HTO 9339, *P. platycephala* HTO 9144, and *S. alata* HTO 9618.

### 4.2. Preparation of Plant Extracts and Phytochemical Analysis

The plant material was collected in the field and brought for further processing at the laboratory. Fresh material was dried at 55 °C for 96 h in a ventilated incubator. Dried leaves of *P. platycephala* and *S. alata* leaves were sieved, weighted, and macerated in 99.5 °GL ethanol for 120 h, *L. leucocephala* was macerated in methanol PA for 48 h and filtered. The filtrates were dried in rotavap (Fisatom, Model 801) to obtain the crude extract (CE). The CE of each plant was partitioned by polarity to separate the hexanic (HexE), acetatic (AcE) and butanolic (BuE) extracts [39].

The phytochemical analysis of crude and partitioned extracts was performed using the method described in Reference [40], in order to verify the presence of secondary metabolic products: phenols, tannins, saponins, flavonoids, and alkaloids.

To characterize the metabolites profile, the extracts with best results in the anthelmintic tests were submitted to analysis in High-Performance Liquid Chromatography (HPLC) (Shimadzu, Tokyo, Japan). The extracts and the standard were prepared in methanol and filtered through a 0.22 μm membrane (Millipore^®^). The separation was carried out by a gradient system, using a reverse-phase Phenomenex Luna 5mm C18 (2) (250 × 4.6mm^2^) column with direct-connect C18 Phenomenex Security Guard Cartridges (4 × 3.0mm^2^). Mobile Phase A was 0.1% phosphoric acid in Milli-Q water and mobile phase B was 0.1% phosphoric acid in Milli-Q water/acetonitrile/methanol (54:35:11). Program gradient: 0 to 2min, 0% B; 2–60 min 100% B. Flow rate: 1mL/min, temperature: 22 °C. UV detection was done at 280 and 254 nm. The compounds were identified by comparing the retention times of samples with standards, such as gallic acid, rutin, ellagic acid, naringin, myricetin, morin, quercetin, naringenin, and kaempferol (Sigma, St. Louis, MO, USA). The amount of each compound was calculated and expressed in micrograms per milligram of extract.

### 4.3. Parasitological Tests

#### Trichostrongylids Eggs and Infective Larvae

Fecal samples were collected from naturally infected sheep. Counting of eggs per gram of feces (EPG) was done using the modified McMaster technique [41]. A pool of fecal samples with EPG higher than 1000 was used for the subsequent tests. Coprocultures were performed according to the technique described by Roberts O’Sullivan [42] using expanded vermiculite as a substrate. The obtained infective stage larvae (L3) were identified as a mixed infection of *Haemonchus* spp., *Trichostrongylus* spp. and *Cooperia* spp., and were used for larval mortality tests.

### 4.4. Egg Hatching Test (EHT)

The EHT followed the method described by Reference [43]. Briefly, 10 g of fresh feces were homogenized with a saturated solution of sodium chloride and passed through a series of overlapping sieves (1000, 100, 55, 29, and 25 µm). The passed material was centrifuged (896G, 5 min), the supernatant was recovered, and followed by 3 times of washing/centrifugation (896G, 5 min) in distilled water (DW) for desalting. After the last centrifugation, the pellet was resuspended in DW with an adjusted concentration of 2 eggs/µL for use in EHT assay. The EHT assay was done in 96 well microplates: 100 µL/well of eggs suspension was distributed and added plant extracts to concentrations of 0.05, 0.1, 0.5, 1.0, and 1.5 mg/mL in an aqueous solution containing 3% of Tween 80. Solutions containing 1% of ivermectin and 3% Tween 80 were used as positive and negative controls respectively. The assay was performed in three replicates, including the negative and positive controls. After an incubation period of 48 h at 26 ± 1 °C, 20µl of Lugol’s iodine solution 3% was added to each well. All eggs and larvae (L1) were counted for each well using an inverted microscope. The eggs’ hatching percentage was determined using the formula: [number of eggs/(number of eggs + number of L1)] ×100. The results from the eggs’ hatching inhibition (EHI) describes the effect of the plant extract added in each test.

### 4.5. Larval Mortality Test (LMT)

LMT was performed according to [44] with tested concentrations of 1.0, 2.5, 5.0 and 7.5 mg/mL of each plant extract. Negative and positive controls were 3% Tween 80 solution and 1% ivermectin respectively. Briefly, third-stage larvae (L3) recovered from coprocultures were washed (3 times) in DW and centrifuged at 2016 G for 10 min. Recovered larvae were submitted to exsheathment using sodium hypochlorite solution (0.0007% v/v). Exsheated larvae were washed in DW three times and the LMT tests were performed in RPMI-1640 medium with 100 UI/mL of penicillin and 100 mg/mL of streptomycin for 24 h at 36 °C. The LMT assay was done in 96 well microplates with an average of 50 larvae per 100 µL of medium. All the assays were completed with three replicates, including the negative and positive controls. The observation and counting of the larvae were done for each well using an inverted microscope. The mortality evaluation was done at 0, 24, 48, and 72 h of incubation to assess the mortality rates for each extract. The effect of the extract in L3 for each group was determined according to the following formula: [alive L3/(alive L3 + dead L3)] ×100.

### 4.6. Statistical Analysis

The results were expressed as a percentage of egg hatching for EHT and percentage of live L3 for LMT after exposure to different concentrations of extracts. The data were analyzed using ANOVA and the means were compared using the Tukey Test (5%) using Graph Prism version 3.0 (GraphPad Software, San Diego, CA, USA). *p*-values of less than 0.05 were considered statistically significant.

### 4.7. Ethics

All the procedures used in this experiment were previously approved by the Ethics Committee on Animal Experimentation of the Federal University of Tocantins, Tocantins State, Brazil (23101.003945-2012-92).

## 5. Conclusions

The evaluation of secondary metabolites from plants with pharmacological potential is important since plants are used in folk medicine for therapeutic purposes, and besides, contribute to the search for new chemical bases. In this preliminary study, based on in vitro results, it is concluded that *P. platycephala* leaves have EHI and larvicidal activity, possibly due to the presence of HT, derivatives of gallic acid, and ellagic acid. The *L. leucocephala* leaves have metabolites with anthelmintic potential that belong to the polyphenols class, possibly CT. The EHI and larvicidal action of *S. alata* seems to be exerted by the synergism of their metabolites.

## Figures and Tables

**Table 1:** Egg hatching inhibition (%) of trichostrongylids eggs (EHI) after 48 exposure to *Leucaena leucocephala, Parkia platycephala* and *Senna alata* extracts (mean ± standard error).

Plants.	1% Ivermectin	3% Tween 80	0.05 mg/mL	0.1 mg/mL	0.5 mg/mL	1.0 mg/mL	1.5 mg/mL
CE *P. platycephala*	100	0 ± 0.0 ^a^	-	0.2 ± 2.3 ^a^	84.5 ± 12.0 ^b^	98.1 ± 0.04 ^b^	99.0 ± 0.0 ^b^
BuE *P. platycephala*	100	27.93 ± 1.56 ^a^	29.26 ± 1.83 ^a^	19.00 ± 2.77 ^a^	60.9 ± 2.67 ^b^	99.6 ± 0.43 ^c^	100 ± 0 ^c^
AcE *P. platycephala*	100	14.36 ± 7.71 ^a^	72.6 ± 3.88 ^b^	71.16 ± 10.83 ^b^	59.8 ± 7.69 ^b^	96.87 ± 3.13 ^c^	100 ± 0 ^c^
CE *L. leucocephala*	100	0 ± 0.0a	-	0.3 ± 10.9 ^a^	90.5 ± 15 ^b^	92.0 ± 3.02 ^b^	93.9 ± 3.5 ^b^
BuE *L. leucocephala*	100	27.93 ± 1.56 ^a^	40.95 ± 0.95 ^ab^	30.94 ± 9.55 ^b^	38.73 ± 9.29 ^ab^	65.42 ± 1.78 ^c^	97.92 ± 2.61 ^d^
HexE *L. leucocephala*	100	14.36 ± 7.71 ^a^	59.4 ± 7.15 ^b^	63.63 ± 4.80 ^b^	66.24 ± 10.68 ^b^	63.64 ± 0.00 ^b^	58.78 ± 8.57 ^b^
AcE *L. leucocephala*	100	26.14 ± 3.07 ^a^	42.41 ± 5.28 ^a^	31.51 ± 4.63 ^a^	48.49 ± 2.42 ^a^	48.93 ± 4.47 ^a^	38.13 ± 5.12 ^a^
CE *S. alata*	100	0 ± 0.0 ^a^	-	0.4 ± 0.2 ^a^	93.4 ± 3.9 ^b^	95.3 ± 2.33 ^b^	99.3 ± 0.3 ^b^
BuE *S. alata*	100	26.14 ± 3.07 ^a^	40.4 ± 6.95 ^a^	39.29 ± 10.71 ^a^	58.27 ± 8.74 ^a^	38.45 ± 11.04 ^a^	41.01 ± 4.86 ^a^
HexE *S. alata*	100	14.36 ± 7.71 ^a^	-	70.0 ± 6.94 ^b^	68.44 ± 7.55 ^b^	62.78 ± 7.22 ^b^	64.62 ± 4.62 ^b^

CE: crude extract; HexE: hexanic extract; AcE: acetatic extract; BuE: butanolic extract; not tested (-). EHI: [number of eggs/(number of eggs + number of L1)] ×100. Different letters in the same line indicate significant differences (*p* < 0.05). Three replicates were done for each sample.

**Table 2:** Mortality rates (%) of third-stage larvae of trichostrongylids after 24, 48, and 72 h exposure to different concentrations of *Parkia platycephala* extract (mean ± standard error).

Concentrations	Evaluation Period			
	0 h	24 h	48 h	72 h
3% Tween 80	4.00 ± 2.0 ^a^	4.00 ± 2.0 ^a^	4.00 ± 2.0 ^a^	4.00 ± 2.0 ^a^
1% Ivermectin	4.00 ± 2.0 ^a^	100 ^b^	100 ^b^	100 ^b^
1.0 mg/mL	4.00 ± 2.0 ^a^	4.51 ± 2.57 ^a^	11.54 ± 2.38 ^a^	3.79 ± 1.04 ^a^
2.5 mg/mL	4.00 ± 2.0 ^a^	2.08 ± 2.08 ^a^	7.68 ± 3.80 ^a^	23.68 ± 0.80 ^b^
5.0 mg/mL	4.00 ± 2.0 ^a^	13.85 ± 1.15 ^a^	9.88 ± 2.35 ^a^	53.19 ± 6.75 ^b^
7.5 mg/mL	4.00 ± 2.0 ^a^	9.09 ± 1.46 ^a^	17.8 ± 1.34 ^a^	54.98 ± 4.20 ^b^

Three replicates were done for each sample. Different letters in the same line indicate significant differences (*p* < 0.05).

**Table 3:** Mortality rates (%) of third-stage larvae of trichostrongylids after 24, 48, and 72 h exposure to different concentrations of *Senna alata* extract (mean ± standard error).

Concentrations	Evaluation Period			
	0 h	24 h	48 h	72 h
3% Tween 80	2.67 ± 2.66 ^a^	2.67 ± 2.66 ^a^	4.67 ± 2.60 ^a^	4.67 ± 2.60 ^a^
1% Ivermectin	2.67 ± 2.66 ^a^	100 ^b^	100 ^b^	100 ^b^
1.0 mg/mL	2.67 ± 2.66 ^a^	8.03 ± 2.75 ^a^	8.03 ± 2.75 ^a^	17.84 ± 8.82 ^a^
2.5 mg/mL	2.67 ± 2.66 ^a^	4.35 ± 4.35 ^a^	49.71 ± 8.61 ^b^	49.58 ± 3.25 ^b^
5.0 mg/mL	2.67 ± 2.66 ^a^	6.36 ± 0.77 ^a^	25.8 ± 4.20 ^b^	47.26 ± 5.70 ^b^
7.5 mg/mL	2.67 ± 2.66 ^a^	2.02 ± 2.08 ^a^	29.4 ± 1.63 ^b^	47.94 ± 5.63 ^b^

Three replicates were done for each sample. Different letters in the same line indicate significant differences (*p* < 0.05).

**Table 4:** Mortality rates (%) of third-stage larvae of trichostrongylids after 24, 48, and 72 h exposure to different concentrations of *Leucaena leucocephala* extract (mean ± standard error).

Concentrations	Evaluation Period			
	0 h	24 h	48 h	72 h
3% Tween 80	4.0 ± 2.0 ^a^	4.0 ± 2.0 ^a^	4.0 ± 2.0 ^a^	4.0 ± 2.0 ^a^
1% Ivermectin	4.0 ± 2.0 ^a^	100^b^	100 ^b^	100 ^b^
1.0 mg/mL	4.0 ± 2.0 ^a^	19.35 ± 5.26 ^a^	7.74 ± 2.97 ^a^	2.62 ± 0.29 ^a^
2.5 mg/mL	4.0 ± 2.0 ^a^	15.35 ± 5.56 ^a^	8.74 ± 3.80 ^a^	5.36 ± 2.24 ^a^
5.0 mg/mL	4.0 ± 2.0 ^a^	12.56 ± 4.17 ^a^	11.25 ± 2.75 ^a^	8.0 ± 4.07 ^a^
7.5 mg/mL	4.0 ± 2.0 ^a^	16.04 ± 3.27 ^a^	24.53 ± 8.4 ^a^	12.6 ± 1.78 ^a^

Three replicates were done for each sample. Different letters in the same line indicate significant differences (*p* < 0.05).
